# Automatic Mode-Matching Method for MEMS Disk Resonator Gyroscopes Based on Virtual Coriolis Force

**DOI:** 10.3390/mi11020210

**Published:** 2020-02-18

**Authors:** Zhihu Ruan, Xukai Ding, Zhengcheng Qin, Jia Jia, Hongsheng Li

**Affiliations:** 1 School of Instrument Science and Engineering, Southeast University, Nanjing 210096, China; 230179760@seu.edu.cn (Z.R.); ding.xk@seu.edu.cn (X.D.); 230189281@seu.edu.cn (Z.Q.); 230169207@seu.edu.cn (J.J.); 2 Key Laboratory of Micro-Inertial Instruments and Advanced Navigation Technology, Ministry of Education, Nanjing 210096, China

**Keywords:** MEMS (Micro-electromechanical Systems) disk resonator gyroscope, frequency split, virtual Coriolis force, electrostatic negative stiffness effect, mode-matching

## Abstract

An automatic mode-matching method for MEMS (Micro-electromechanical Systems) disk resonator gyroscopes (DRGs) based on virtual Coriolis force is presented in this paper. For this mode-matching method, the additional tuning electrodes are not required to be designed, which simplifies the structure design. By using the quadratic relationship between the driving voltage and the electrostatic force, the virtual Coriolis force is obtained by applying an AC voltage whose frequency is half of the driving mode resonant frequency to the sense electrode. The phase difference between the virtual Coriolis force and the sense output signal is used for mode-matching. The structural characteristics and electrode distribution of the DRG are briefly introduced. Moreover, the mode-matching theories of the DRG are studied in detail. The scheme of the mode-matching control system is proposed. Simultaneously, the feasibility and effectiveness of the mode-matching method are verified by system simulation. The experimental results show that under the control of mode-matching at room temperature, the bias instability is reduced from 30.7575 ${}^{{}^{\circ}}$/h to 2.8331 ${}^{{}^{\circ}}$/h, and the Angle Random Walk (ARW) decreases from 1.0208 ${}^{{}^{\circ}}/\sqrt{h}$ to 0.0524 ${}^{{}^{\circ}}/\sqrt{h}$. Compared with the mode mismatch condition, the ARW is improved by 19.48 times.

## 1. Introduction

Coriolis vibratory gyroscopes with integrated Micro-electromechanical Systems (MEMS) are widely used in automotive, consumer electronics, industrial, aerospace, and other fields due to their small size, low power consumption, and low cost [1]. The most common high-performance vibration gyroscopes are based on the energy transfer caused by the Coriolis coupling between a pair of degenerate vibration modes associated with an axisymmetric structure. Common examples include hemispherical shells [2,3,4], rings [5,6,7,8], and disks [9,10,11,12]. The disk resonator gyroscope (DRG) is a kind of high-performance MEMS gyroscope that has attracted much attention in recent years [13,14]. It adopts symmetrical nested ring structures and a single-center anchorage. It has the advantages of high thermal stability, low anchorage loss, large modal mass, and an abundant number of internal and external axial electrodes [15,16,17]. Due to manufacturing defects, it is difficult to perfectly match the resonance frequencies of the two modes, which results in a frequency split. The frequency split degrades the energy conversion efficiency of Coriolis force, which further results in degraded sensitivity, resolution, signal-to-noise ratio (SNR), and poor bias stability [18].

Therefore, the mode-matching technology improves the bias stability and mechanical sensitivity of MEMS gyroscopes by eliminating the frequency split between the driving mode and the sensing mode, which has attracted significant attention from researchers [19,20,21]. Furthermore, some frequency modification techniques are proposed. Laser finishing, ion beam milling, and selective deposition mass loading reduce frequency split [22,23]. However, these methods are often used to change the dynamic characteristics of the sensor permanently, and this type of tuning is inflexible and time-consuming. The tuning control is limited and can only be achieved offline.

Electrostatic tuning is usually used for mode-matching, which can provide more flexibility and real-time realization [24,25,26,27,28,29]. The mode-matching process based on phase-locked loop (PLL) takes advantage of the phase delay of $90^{{}^{\circ}}$ between the quadrature input and output of the sensing mode. This feature is used in [27] to achieve mode-matching and to adjust the tuning voltage using PLL technology. The amplitude-frequency characteristic refers to the maximum amplitude of the quadrature response signal during mode-matching [28]. However, in these methods, the signal from the Coriolis demodulation channel is used to control the frequency tuning voltage. Therefore, the angular velocity can only be measured if the tuning voltage is fixed, and the matching loop is disconnected after mode-matching. Thus, these methods cannot achieve real-time mode-matching. However, in practical applications, the frequency split of the vibratory mode changes with changes in environmental parameters [29]. Therefore, such one-time matching methods are not enough.

A real-time mode-matching method without affecting normal angular rate detection is urgently needed [30,31,32,33,34]. A mode-matching force balance control method for a MEMS vibrating gyroscope was proposed in [31], making the frequency split 20 mHz during modal matching. However, it is worth noting that force-to-rebalance (FTR) closed-loop mode tuning may be difficult to achieve without using a rate table because the driving electrode of sense mode is already used for FTR control. An automatic mode-matching control structure is introduced in [32]. However, the influence of quadrature error on mode-matching is not discussed in this paper, nor is the suppressed orthogonal error. A utility algorithm to reduce frequency mismatch via stiffness perturbation is introduced in [33], but this tuning method is currently implemented offline. A proportional driving force to the sensor electrode and designs a frequency-tuned loop is introduced in [34]. However, it does not take into account that when the gyroscope is working normally, the driving force is adjusted in real-time, which results in low matching accuracy. At the same time, the sensor output bias signal is also variable, affecting bias stability. Other [35,36,37] mode-matching methods are based on the amplitude-frequency or phase-frequency characteristics of the quadrature response. They require a small number of quadrature signals to detect the magnitude of the quadrature or the phase difference between the quadrature and the Coriolis force output. However, these methods are not effective when quadrature needs to be suppressed. Recently, new frequency tuning techniques have also been reported, such as structure stiffening [38,39] and softening [39] by using phase-change materials. Currently, they are mainly used in vanadium dioxide (${\mathrm{VO}}_{2}$) based MEMS resonators.

A real-time mode-matching technique for MEMS DRG based on virtual Coriolis force is proposed. The additional independent tuning electrode is unnecessary to be added, which simplifies the structure design. The paper is organized as follows. Section 2 introduces the microstructure of the disk resonator, establishes a mathematical model, derives the electrostatic force equation, and discusses the effect of negative stiffness of the electrostatic force. In Section 3, the principle of the mode-matching control system framework is analyzed in detail. Section 4 gives the system simulation analysis. Section 5 provides experimental results, validates theoretical analysis, and demonstrates the benefits of mode-matching, primarily in terms of noise performance and bias instability. Finally, Section 6 provides a summary of the full text.

## 2. Architecture and Motion Model of DRG

### 2.1. Basic Structure of DRG

**Figure 1:**
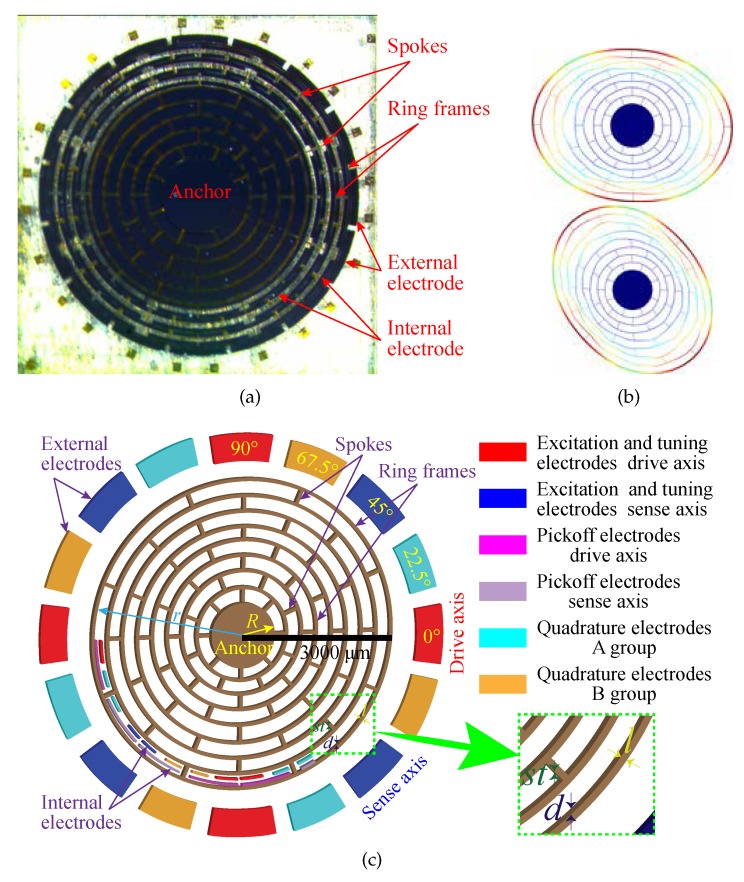
Microscope image, mode shapes and microstructure schematic of DRG: (**a**) microscope images of DRG; (**b**) mode shapes of the n=2 wine-glass modes of DRG; (**c**) the 3D schematic of the microstructure of the DRG.

**Figure 2:**
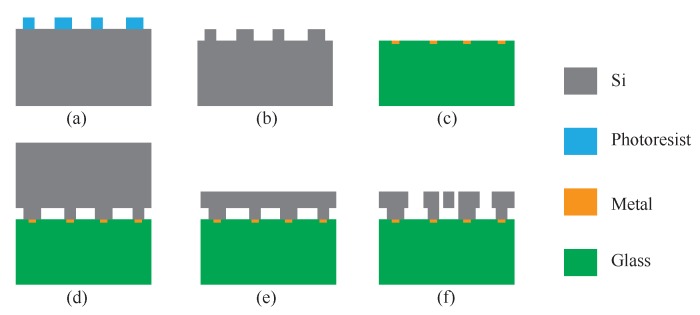
The fabrication process of the DRG: (**a**) photo-etching; (**b**) bonding area etching; (**c**) metal deposition; (**d**) anodic bonding; (**e**) thinning and polishing; (**f**) dry etching and structure release.

In this paper, the microscope image, the mode shapes, and the 3D schematic of the microstructure of the DRG are shown in Figure 1. As shown in Figure 1a,c, the disk resonator consists of eight concentric nested rings connected by spokes. The disk resonator is suspended from a single-center anchor. The disk resonator is surrounded by 16 external electrodes for excitation, frequency tuning, and quadrature null. These arc-shaped grooves can be used to make internal electrodes. In Figure 1c, the internal electrodes in several grooves are shown. The internal electrodes of the other grooves are designed according to the same arrangement rule. The internal electrodes consist of two parts; one is used as the pickoff electrodes (the internal pickoff electrodes with the same function increase the area through internal interconnection), the other is used as the excitation electrodes. Internal and external excitation electrodes increase the area through interconnection. Nested ring thickness, disk radius, anchoring radius, nested ring width, and spoke thickness are defined as *d*, *r*, *R*, *l*, and st, respectively. The capacitive air-gap formed between the surface of each nested ring of the disk resonator and electrode is $h_{0}$, and ϑ is the width of a single electrode. The fabrication process is shown in Figure 2. A more detailed description of the fabrication process can be found in our previous article [18].

For the rate mode of the DRG, one of the two orthogonal modes is driven into a stable resonant state, so it is usually called the driving mode. Another mode senses the input angular velocity through Coriolis coupling, which is called the sensing mode. Ideally, the two modes have the same natural frequency. The disk resonator is excited in the elliptical driving mode of n=2. The Coriolis force is detected in the degenerate sensing mode. The geometric distance between the degenerate sensing mode and the driving mode is 45${}^{{}^{\circ}}$, as shown in Figure 1b. When the electrostatic driving force is applied in the $0^{{}^{\circ}}$ (*x*-direction), the vibration disk resonator will flex along the *x*-direction with a fixed amplitude. Because of the Coriolis effect, the vibratory disk will bend at $45^{{}^{\circ}}$ (*y*-direction), the amplitude of which is proportional to the input angular velocity.

### 2.2. Dynamic Model of DRG

Neglecting the influence of centrifugal force and angular acceleration, a simplified equation of motion for the plane bending vibration of n=2 operating mode can be established as a generalized two-dimensional oscillator with mass, damping and stiffness defects. The motion Equation of the non-ideal DRG is described as [19,20,40,41]

(1) $$\left[M\right]\ddot{P}+\left([C]+[M][G]\right)\dot{P}+\left[K\right]P=F,$$

where [M], [C], [K], and [G] are mass, damping, stiffness matrices, and gyroscopic effect matrices respectively. These matrices are described in detail as

$$\begin{array}{c} \left[M\right]=m_{0}\left[\sigma \right],\;\left[C\right]=c_{0}\left[\delta \right],\;\left[K\right]=k_{0}\left[\mu \right], \end{array}$$

with

$$\begin{array}{c} \left[\sigma \right]=\left[\begin{array}{cc} 1+\sigma_{1} & \sigma_{2}\\ \sigma_{2} & 1-\sigma_{1} \end{array}\right],\;\left[\delta \right]=\left[\begin{array}{cc} 1+\delta_{1} & \delta_{2}\\ \delta_{2} & 1-\delta_{1} \end{array}\right],\;\left[\mu \right]=\left[\begin{array}{cc} 1+\mu_{1} & \mu_{2}\\ \mu_{2} & 1-\mu_{1} \end{array}\right],\\ {}[G]=2\left[\begin{array}{cc} 0 & -\nu \Omega \\ \nu \Omega & 0 \end{array}\right],\;P={\left[\begin{array}{cc} x & y \end{array}\right]}^{T},\;F={\left[\begin{array}{cc} F_{x} & F_{y} \end{array}\right]}^{T}, \end{array}$$

where ν is the angular gain factor; $m_{0}$ is the generalized equivalent mass of perfect disk resonator; $c_{0}$ is the damping coefficient of perfect disk resonator; $k_{0}$ is the stiffness coefficient of perfect disk resonator; P is displacement vector; *F* both the electrostatic forces and the electrostatic stiffness effects, which will be discussed and analyzed in detail in Section 2.3; σ, δ, μ are the mass, damping and stiffness perturbations from the ideal DRG, which are usually very small parameter values; Ω is the input angular rate of the DRG.

The Equations of motion (1) can be rewritten as

(2) $$\ddot{P}+\left(\frac{2}{\tau_{0}}{\left[\sigma \right]}^{-1}\left[\delta \right]+\left[G\right]\right)\dot{P}+\omega_{0}^{2}{\left[\sigma \right]}^{-1}\left[\mu \right]P=\frac{1}{m_{0}}{\left[\sigma \right]}^{-1}F,$$

where $\omega_{0}$ ($\omega_{0}=\sqrt{k_{0}/m_{0}}$) is the natural frequency of perfect disk resonator; $\tau_{0}$ ($\tau_{0}=2Q_{0}/\omega_{0}$) is attenuation time constant corresponding to $\omega_{0}$; $Q_{0}$ $\left(Q_{0}=\omega_{0}m_{0}/c_{0}\right)$ is the quality factor of the disk resonator.

Moreover, if the second-order perturbed infinitesimal parameters are ignored, the following formula can be expressed as

(3) $$\begin{array}{cc} {\left[\sigma \right]}^{-1} & \approx \left[\begin{array}{cc} 1-\sigma_{1} & -\sigma_{2}\\ -\sigma_{2} & 1+\sigma_{1} \end{array}\right], \end{array}$$

(4) $$\begin{array}{cc} \omega_{0}^{2}{\left[\sigma \right]}^{-1}\left[\mu \right] & ≜\left[\begin{array}{cc} \omega_{xx}^{2} & \omega_{xy}^{2}\\ \omega_{yx}^{2} & \omega_{yy}^{2} \end{array}\right]\approx \omega_{0}^{2}\left[\begin{array}{cc} 1-\sigma_{1}+\mu_{1} & \mu_{2}-\sigma_{2}\\ \mu_{2}-\sigma_{2} & 1+\sigma_{1}-\mu_{1} \end{array}\right],\;\omega_{xy}^{2}=\omega_{yx}^{2}, \end{array}$$

(5) $$\begin{array}{cc} \frac{2}{\tau_{0}}{\left[\sigma \right]}^{-1}\left[\delta \right] & ≜\left[\begin{array}{cc} \frac{2}{\tau_{xx}} & \frac{2}{\tau_{xy}}\\ \frac{2}{\tau_{yx}} & \frac{2}{\tau_{yy}} \end{array}\right]\approx \frac{2}{\tau_{0}}\left[\begin{array}{cc} 1-\sigma_{1}+\delta_{1} & \delta_{2}-\sigma_{2}\\ \delta_{2}-\sigma_{2} & 1+\sigma_{1}-\delta_{1} \end{array}\right],\;\frac{2}{\tau_{xy}}=\frac{2}{\tau_{yx}}. \end{array}$$

The influence of damping on the resonance frequency can be ignored. Here, damping is assumed to be 0, so that the characteristics of frequency difference can be better analyzed. Then, without considering the influence of the electrostatic negative stiffness on the frequency, combining Equation (4), Equation (2) is transformed into the first-order state-space expression reflecting the vibration displacement and velocity of the disk resonator. Therefore, the state space expression can be expressed as

(6) $$\dot{Z}=AZ+BU,$$

where

(7) $$\begin{array}{cc} A & =\left[\begin{array}{cccc} 0 & 0 & 1 & 0\\ 0 & 0 & 0 & 1\\ -\omega_{0}^{2}\left(1-\sigma_{1}+\mu_{1}\right) & -\omega_{0}^{2}\left(\mu_{2}-\sigma_{2}\right) & 0 & 2\nu \Omega \\ -\omega_{0}^{2}\left(\mu_{2}-\sigma_{2}\right) & -\omega_{0}^{2}\left(1+\sigma_{1}-\mu_{1}\right) & -2\nu \Omega & 0 \end{array}\right],\\ B & =\frac{1}{m_{0}}\left[\begin{array}{cc} 0 & 0\\ 0 & 0\\ 1-\sigma_{1} & -\sigma_{2}\\ -\sigma_{2} & 1+\sigma_{1} \end{array}\right],\;Z=\left[\begin{array}{c} x\\ y\\ \dot{x}\\ \dot{y} \end{array}\right],\;U=\left[\begin{array}{c} f_{x}\\ f_{y} \end{array}\right]. \end{array}$$

The two imaginary parts of the eigenvalues of matrix [A] are two modal principal frequencies of the disk resonator [42,43]. It is advisable to set the form of the eigenvalue of matrix [A] as $\pm i\omega_{1}$, $\pm i\omega_{2}$. In practice, Ω≪ω, accordingly, the influence of the input angular velocity Ω on the modal frequency is ignored, so the expressions of modal principal frequencies for DRG are

(8) $$\begin{array}{cc} \omega_{1} & =\sqrt{2\nu^{2}\Omega^{2}+\omega_{0}^{2}\left(1-\sqrt{{\left(\mu_{1}-\sigma_{1}\right)}^{2}+{\left(\mu_{2}-\sigma_{2}\right)}^{2}+\frac{4\nu^{4}\Omega^{4}}{\omega_{0}^{4}}+\frac{4\nu^{2}\Omega^{2}}{\omega_{0}^{2}}}\right)},\\ \; & \approx \omega_{0}\sqrt{1-\sqrt{{\left(\mu_{1}-\sigma_{1}\right)}^{2}+{\left(\mu_{2}-\sigma_{2}\right)}^{2}}},\\ \omega_{2} & =\sqrt{2\nu^{2}\Omega^{2}+\omega_{0}^{2}\left(1+\sqrt{{\left(\mu_{1}-\sigma_{1}\right)}^{2}+{\left(\mu_{2}-\sigma_{2}\right)}^{2}+\frac{4\nu^{4}\Omega^{4}}{\omega_{0}^{4}}+\frac{4\nu^{2}\Omega^{2}}{\omega_{0}^{2}}}\right)},\\ \; & \approx \omega_{0}\sqrt{1+\sqrt{{\left(\mu_{1}-\sigma_{1}\right)}^{2}+{\left(\mu_{2}-\sigma_{2}\right)}^{2}}}, \end{array}$$

where $\omega_{1}$, $\omega_{2}$ are the resonance frequencies of the frequency principal axes, which can reach the maximum and minimum values respectively. Moreover, it can be seen from Equation (8) that the frequency error is mainly affected by mass perturbation and stiffness perturbation coefficient. Assuming $\omega_{1}<\omega_{2}$, then the frequency split between the two modes can be expressed as $\Delta \omega =\omega_{2}-\omega_{1}$.

### 2.3. Electrostatic Force and Electrostatic Negative Stiffness Effect

**Figure 3:**
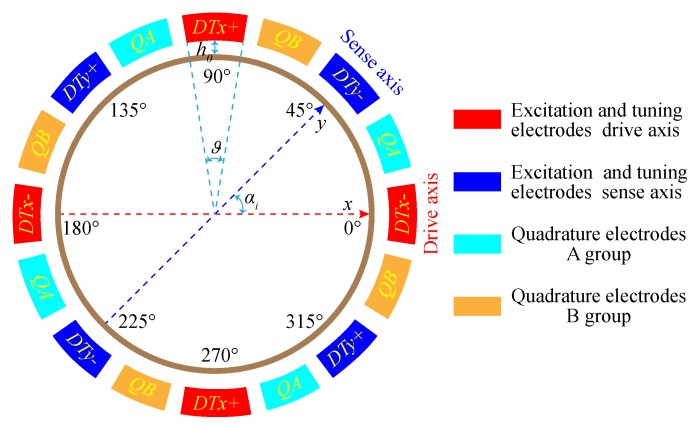
Distribution schematic of 16 external electrodes of the DRG.

As shown in Figure 1a,c, the internal electrodes of the disk resonator are used as the vibration information pickoff. The external electrodes are used as excitation (excitation and tuning act on the same electrode) and quadrature stiffness correction, respectively. Figure 3 shows a schematic of the symmetrical and differential configuration of DRG with sixteen external electrodes. The “+” and “−” symbols in Figure 3 represent the in-phase and anti-phase relations of the driving signal of the n=2 working mode.

In general, the radial displacement of the disk resonator vibration is much less than $h_{0}$, so the potential energy stored in the capacitor formed between the outer surface of the disk resonator. Any electrode can be expressed in a convenient form by Taylor series expansion, and the term larger than ${\left(u/h_{0}\right)}^{3}$ is ignored. Therefore, combining Table 1, the potential energy can be expressed as [19,44]

(9) $$E_{c}=\frac{\epsilon rd}{2h_{0}}{V_{i}}^{2}\int_{\alpha_{i}-\frac{\vartheta }{2}}^{\alpha_{i}+\frac{\vartheta }{2}}\left[1+\frac{u}{h_{0}}+\left(\frac{u^{2}}{{h_{0}}^{2}}\right)+\left(\frac{u^{3}}{{h_{0}}^{3}}\right)+\cdots \right]\;d\theta ,$$

where *u* (u=xcosnθ+ysinnθ) is radial displacement of disk resonator; θ is the azimuth; n=2 is the working mode, the following definition of *n* is the same; $\alpha_{i}$ is the angle between the central axis of the electrode and the *x*-axis in Figure 3; $\epsilon =8.85\times 10^{-12}$ F/m is the permittivity in vacuum; $V_{i}$ is the driving voltage, which is usually set to an AC signal biased by a DC voltage; *r*, $h_{0}$, and *d* are as those defined in Section 2.1.

According to Equation (9) and the electrostatic force formula of the planar capacitor, the electrostatic force between the single excitation electrode and the outer surface of the disk resonator can be obtained as

(10) $$f≜\left[\begin{array}{c} f_{x}\\ f_{y} \end{array}\right]=\lambda \left[\overset{V_{i}^{2}}{\overset{︷}{\underset{\mathrm{DC}\;\mathrm{Static}\;\mathrm{Force}}{\underset{︸}{\left(V_{dc}^{2}+\frac{1}{2}v_{ac}^{2}\right)}}\pm \underset{\omega_{d}\;\mathrm{Electrostatic}\;\mathrm{Force}}{\underset{︸}{2V_{dc}v_{ac}\cos\left(\omega_{d}t\right)}}+\underset{2\omega_{d}\;\mathrm{Electrostatic}\;\mathrm{Force}}{\underset{︸}{\frac{1}{2}v_{ac}^{2}\cos\left(2\omega_{d}t\right)}}}}\right]\left[\begin{array}{c} \cos(2\alpha_{i})\\ \sin(2\alpha_{i}) \end{array}\right],$$

with

$$\lambda =\frac{\epsilon rd}{2h_{0}^{2}}\sin\left(\vartheta \right),$$

where $\alpha_{i}=0,\pi /4,\pi /2,3\pi /4,\pi ,5\pi /4,3\pi /2,7\pi /4$, in that way, $\cos(4\alpha_{i})=\pm 1$ and $\sin(4\alpha_{i})=0$ for all 8 values of $\alpha_{i}$; $\omega_{d}$ is the angular frequency of excitation AC voltage. The excitation electrodes of the driving mode are located at $\alpha_{i}=0,\pi /2,\pi ,3\pi /2$. The excitation electrodes of the sensing mode are located at $\alpha_{i}=\pi /4,3\pi /4,5\pi /4,7\pi /4$.

It can be seen in Equation (10) that the voltage $V_{i}$ ($V_{x}$ or $V_{y}$) applied to the excitation electrode plates will excite three electrostatic forces on the disk resonator. Among them, the first term is a constant value component, which only affects the static displacement of the harmonic oscillator. The second term is an AC electrostatic force signal with angular frequency $\omega_{d}$. Its amplitude is determined by $V_{dc}$ and $v_{ac}$. The third term is also the AC electrostatic force signal with angular frequency $2\omega_{d}$. Its amplitude can only be altered by changing the input $v_{ac}$ value. The latter two parts can be used to excite the vibration state of the disk resonator. If the second part is selected for control, the input frequency needs to be $\omega_{d}=\omega_{0}$. If the third part is used for control, the input frequency $\omega_{d}=\frac{1}{2}\omega_{0}$. Either way can be chosen, but the difference in amplitude and phase should be noted [45].

Furthermore, according to Equations (9) and (10), it can be seen that the excitation voltage on the excitation electrode will also introduce the electrostatic negative stiffness effect. The electrostatic stiffness matrix generated by the excitation voltage is

(11) $$\left[K_{T}\right]=\left[\begin{array}{cc} \frac{\partial^{2}E_{c}}{\partial x^{2}} & \frac{\partial^{2}E_{c}}{\partial x\partial y}\\ \frac{\partial^{2}E_{c}}{\partial y\partial x} & \frac{\partial^{2}E_{c}}{\partial y^{2}} \end{array}\right]=\frac{\epsilon rd}{2h_{0}^{3}}V_{i}^{2}\left[\begin{array}{cc} \vartheta +\frac{1}{n}\cos\left(2n\alpha_{i}\right)\;\cdot \;\sin\left(n\vartheta \right) & \frac{1}{2n}\sin\left(2n\alpha_{i}\right)\;\cdot \;\sin\left(n\vartheta \right)\\ \frac{1}{2n}\sin\left(2n\alpha_{i}\right)\;\cdot \;\sin\left(n\vartheta \right) & \vartheta -\frac{1}{n}\cos\left(2n\alpha_{i}\right)\;\cdot \;\sin\left(n\vartheta \right) \end{array}\right],$$

where n=2.

Then, since the AC voltage term of the excitation voltage $V_{x}$ or $V_{y}$ alternates rapidly with time, its average effect on the stiffness of the DRG is approximately 0. Because ϑ value is small, the sin(2ϑ)≈2ϑ, the Equation is further simplified to be available as

(12) $$\left[K_{T}\right]\approx \kappa \vartheta \left[\begin{array}{cc} 2V_{x}^{2} & 0\\ 0 & 2V_{y}^{2} \end{array}\right]\approx \left[\begin{array}{cc} 2V_{dx}^{2} & 0\\ 0 & 2V_{dy}^{2} \end{array}\right],$$

where

$$\begin{array}{cc} V_{dx}^{2} & =V_{dcx}^{2}+\frac{1}{2}v_{acx}^{2},\\ V_{dy}^{2} & =V_{dcy}^{2}+\frac{1}{2}v_{acy}^{2}. \end{array}$$

where $\kappa =\frac{\epsilon rd}{2h_{0}^{3}}$, it is assumed that $V_{x}=V_{dcx}\pm v_{acx}\cos\left(\omega_{d}t\right)$ and $V_{y}=V_{dcy}\pm v_{acy}\cos\left(\omega_{d}t\right)$ is the excitation voltages of the driving mode and the sensing mode, respectively.

Equation (12) shows that the $V_{dx}$ generates electrostatic stiffness along the *y*-axis is approximately 0, and the same is true of $V_{dy}$. Furthermore, the corresponding axial stiffness can be softened by adjusting $V_{dx}$ or $V_{dy}$ independently. Moreover, it also shows that the electrostatic coupling stiffness between the two axes will not be generated when the voltages are applied to the excitation electrodes.

**Figure 4:**
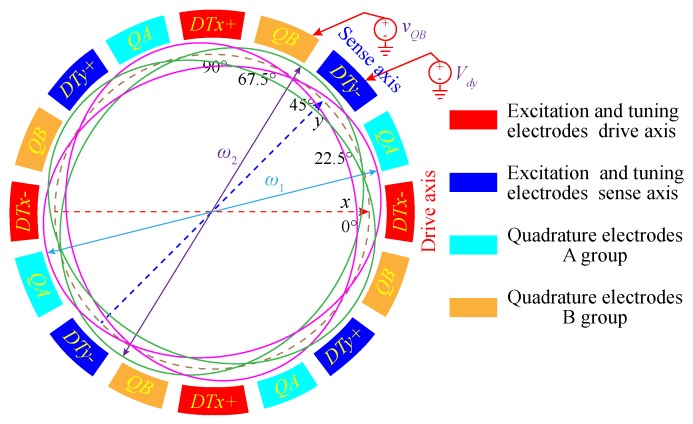
Schematic diagram of the tuning electrodes of the non-ideal DRG.

Similarly, according to Equations (9)–(11), applying DC voltages to all quadrature correction electrodes, as shown in Figure 4, will not only generate static forces but also introduce an electrostatic stiffness effect. From the above analysis, it will only affect the static displacement of the disk resonator vibration. The electrostatic stiffness matrix is

(13) $$\left[K_{Q}\right]\approx \kappa \vartheta \left[\begin{array}{cc} \left(V_{QA}^{2}+V_{QB}^{2}\right) & \frac{1}{2}\left(V_{QA}^{2}-V_{QB}^{2}\right)\\ \frac{1}{2}\left(V_{QA}^{2}-V_{QB}^{2}\right) & \left(V_{QA}^{2}+V_{QB}^{2}\right) \end{array}\right],$$

where $V_{QA}$ and $V_{QB}$ are DC voltages applied to two quadrature correction electrodes of A and B groups. In general, according to the positive and negative coupling stiffness of the resonator structure, a correction voltage can be applied to one of the two sets of quadrature correction electrodes, and the other can be connected to the ground.

Besides, it is easy to verify that the equivalent force exerted by the differential electrode configuration on the DRG in Figure 3 is

(14) $$F=2f+[K_{E}]P.$$

where $\left[K_{E}\right]=2\left[K_{T}\right]+\left[K_{Q}\right]$.

It can be known from Equations (12) and (13) that the excitation voltages will generate electrostatic stiffness along the excitation directions. In contrast, the quadrature control voltages will cause both the electrostatic stiffness along the two excitation directions and the coupling stiffness between the two excitation directions. The influences of $\left[K_{T}\right]$ and $\left[K_{Q}\right]$ on the operating mode of the resonator will be discussed in detail in Section 3.

## 3. Automatic Mode-Matching method based on Virtual Coriolis Force

### 3.1. Electrostatic Stiffness Tuning Theory

Considering that the mass and stiffness perturbations of the resonator in this paper are extremely small, the second-order infinitesimals can be ignored. By substituting Equation (14) into Equation (2), the dynamic model of the DRG under the electrostatic negative stiffness effect can be obtained as

(15) $$\ddot{P}+\left(\frac{2}{\tau_{0}}{\left[\sigma \right]}^{-1}\left[\delta \right]+\left[G\right]\right)\dot{P}+\left[{\tilde{\omega }}_{E}^{2}\right]P=\frac{1}{m_{0}}{\left[\sigma \right]}^{-1}f_{d},$$

where

(16) $$\begin{array}{cc} \left[{\tilde{\omega }}_{E}^{2}\right] & ≜\left[\begin{array}{cc} {\tilde{\omega }}_{xx}^{2} & {\tilde{\omega }}_{xy}^{2}\\ {\tilde{\omega }}_{yx}^{2} & {\tilde{\omega }}_{yy}^{2} \end{array}\right]\\ \; & =\omega_{0}^{2}{\left[\sigma \right]}^{-1}\left[\mu \right]-\frac{1}{m_{0}}{\left[\sigma \right]}^{-1}\left[K_{E}\right]\\ \; & \approx \omega_{0}^{2}\left[\begin{array}{cc} 1-\sigma_{1}+\mu_{1}-\gamma_{11} & \mu_{2}-\sigma_{2}-\gamma_{12}\\ \mu_{2}-\sigma_{2}-\gamma_{21} & 1+\sigma_{1}-\mu_{1}-\gamma_{22} \end{array}\right],\\ \gamma_{11} & =\frac{\kappa \vartheta }{m_{0}\omega_{0}^{2}}\left(4V_{dx}^{2}+V_{QA}^{2}+V_{QB}^{2}\right),\;\gamma_{22}=\frac{\kappa \vartheta }{m_{0}\omega_{0}^{2}}\left(4V_{dy}^{2}+V_{QA}^{2}+V_{QB}^{2}\right),\\ \gamma_{12} & =\gamma_{21}=\frac{\kappa \vartheta }{2m_{0}\omega_{0}^{2}}\left(V_{QA}^{2}-V_{QB}^{2}\right),\;{\tilde{\omega }}_{xy}^{2}={\tilde{\omega }}_{yx}^{2},\;f_{d}=\left[\begin{array}{c} 2f_{x}\\ 2f_{y} \end{array}\right]. \end{array}$$

Equation (15) illustrates that the electrostatic negative stiffness effect softens the two modal frequencies. Likewise, by using the characteristic equation, two modal frequencies under the action of electrostatic stiffness can be obtained as

(17) $$\begin{array}{cc} {\tilde{\omega }}_{1} & =\omega_{0}\sqrt{1-\frac{\gamma_{11}+\gamma_{22}}{2}-\sqrt{{\left[\left(\mu_{1}-\sigma_{1}\right)-\frac{\gamma_{11}-\gamma_{22}}{2}\right]}^{2}+{\left(\mu_{2}-\sigma_{2}-\gamma_{12}\right)}^{2}}}, \end{array}$$

(18) $$\begin{array}{cc} {\tilde{\omega }}_{2} & =\omega_{0}\sqrt{1-\frac{\gamma_{11}+\gamma_{22}}{2}+\sqrt{{\left[\left(\mu_{1}-\sigma_{1}\right)-\frac{\gamma_{11}-\gamma_{22}}{2}\right]}^{2}+{\left(\mu_{2}-\sigma_{2}-\gamma_{12}\right)}^{2}}}, \end{array}$$

where ${\tilde{\omega }}_{1}$ and ${\tilde{\omega }}_{2}$ (${\tilde{\omega }}_{1}<{\tilde{\omega }}_{2}$) are the resonance frequencies of two modal frequencies principal axes under the action of electrostatic negative stiffness. Generally, when the DRG works in the rate mode, ${\tilde{\omega }}_{1}$ is the angular frequency of the driving mode, and ${\tilde{\omega }}_{2}$ is the angular frequency of the sensing mode.

Further, combining Equations (17) and (18), the relation between the frequency difference and the quadrature control voltage and the tuning voltage can be obtained as

(19) $${\tilde{\omega }}_{2}^{2}-{\tilde{\omega }}_{1}^{2}=2\omega_{0}^{2}\sqrt{{\left[\left(\mu_{1}-\sigma_{1}\right)-\frac{\gamma_{11}-\gamma_{22}}{2}\right]}^{2}+{\left(\mu_{2}-\sigma_{2}-\gamma_{12}\right)}^{2}}.$$

The conditions of the mode-matching are given as

(20) $$\begin{array}{cc} \mu_{1}-\sigma_{1} & =\frac{1}{2}\left(\gamma_{11}-\gamma_{22}\right), \end{array}$$

(21) $$\begin{array}{cc} \mu_{2}-\sigma_{2} & =\gamma_{12}. \end{array}$$

Anti-angle term (coupling term) of frequency matrix can be eliminated by using $\gamma_{12}$ alone in Equation (16), which means that the voltage difference applied to the quadrature control electrodes can electrostatically correct the quadrature coupling so that the two modal principal axes of the working mode are aligned with each corresponding excitation electrode. Besides, it can also be seen from Equations (20) and (21) that in order to achieve mode-matching, the quadrature error of the DRG must be restrained first. Otherwise, the frequency split cannot be eliminated.

As shown in Figure 4, if the azimuth angle of $\omega_{2}$ ($\omega_{2}>\omega_{1}$) is between the *y*-direction electrodes and the quadrature correction B electrodes, these two groups of electrodes should be applied with appropriate DC voltages to achieve mode-matching. Therefore, after the stiffness coupling term is eliminated by adjusting the quadrature stiffness correction voltage, by using the characteristic equation, the two modal frequencies of the DRG under quadrature stiffness correction can be obtained as

(22) $$\begin{array}{cc} {\tilde{\omega }}_{m1} & ={\tilde{\omega }}_{xx}=\omega_{0}\sqrt{1+\mu_{1}-\sigma_{1}-\gamma_{11}}, \end{array}$$

(23) $$\begin{array}{cc} {\tilde{\omega }}_{m2} & ={\tilde{\omega }}_{yy}=\omega_{0}\sqrt{1-\mu_{1}+\sigma_{1}-\gamma_{22}}. \end{array}$$

Therefore, by using the electrostatic negative stiffness effect, the frequency matching is realized by adjusting the voltages on the excitation electrodes of *x* and *y* axes. When the DRG is working normally, the driving excitation electrodes, the sensing excitation electrodes and the quadrature stiffness correction electrodes all have corresponding DC bias voltages. When the modes match $\left(\gamma_{11}-\gamma_{22}=2\left(\mu_{1}-\sigma_{1}\right)\right)$, the two modal frequencies of the DRG under electrostatic stiffness tuning are

(24) $${\tilde{\omega }}_{m1}={\tilde{\omega }}_{m2}={\tilde{\omega }}_{0}=\omega_{0}\sqrt{1-\frac{\gamma_{11}+\gamma_{22}}{2}}.$$

### 3.2. Mode-Matching Control System

#### 3.2.1. System Features in Mode-Matching

The driving mode control system adopts the traditional PLL + AGC (automatic gain control) methods to keep the drive pick off amplitude stable and work on its resonant frequency. When the quadrature stiffness correction voltage $V_{QA}=V_{QB}=0$ ($\gamma_{12}=\gamma_{21}=0$), and the mode mismatch exists, then, according to Equations (15) and (16), the harmonic vibration of sensing mode (*y*-axis) of the DRG can be expressed as

(25) $$\ddot{y}+\frac{2}{\tau_{yy}}\dot{y}+{\tilde{\omega }}_{yy}^{2}y=\underset{g_{\Omega }}{\underset{︸}{-2\nu \Omega \dot{x}}}-\underset{g_{\tau_{yx}}}{\underset{︸}{\frac{2}{\tau_{yx}}\dot{x}}}-\underset{g_{q}}{\underset{︸}{{\tilde{\omega }}_{yx}^{2}x}}-\underset{g_{xc}}{\underset{︸}{\frac{2\sigma_{2}}{m_{0}}f_{x}}}+\underset{g_{y}}{\underset{︸}{\frac{2(1+\sigma_{1})}{m_{0}}f_{y}}},$$

where $\Omega =\Omega_{c}\cos\left(\omega_{\Omega }t\right)$ is the angular rate; $g_{\Omega }$ is the Coriolis acceleration; $g_{\tau_{yx}}$ is the damping coupling acceleration; $g_{xc}$ is excitation coupling acceleration; $g_{q}$ is the quadrature stiffness coupling acceleration; $f_{y}$ is the excitation force of *y*-axis, and $f_{y}=0$, $g_{y}$ is the excitation acceleration; ${\tilde{\omega }}_{yy}^{2}=\omega_{yy}^{2}-\eta V_{tyy}^{2}$; $\eta =\frac{\kappa \vartheta }{m_{0}}$; $V_{tyy}^{2}=\left(4V_{dy}^{2}+V_{QA}^{2}+V_{QB}^{2}\right)$; $V_{QA}=0$.

The excitation voltage $V_{x}=V_{dcx}\pm v_{acx}\cos\left(\omega_{d}t\right)$ is applied to the corresponding excitation driving electrodes, and $V_{dcx}\gg v_{acx}$. Then $V_{x}$ is substituted into Equation (10), ignoring the DC static force and the electrostatic AC force of $2\omega_{d}$ to obtain the driving force $f_{x}$. When the DRG is operated in rate mode, the electrostatic driving force $f_{x}=A_{fx}\cos\left(\omega_{d}t\right)$ excites the driving mode to resonance with a constant amplitude $A_{x}$. $A_{fx}$ is the amplitude of the electrostatic driving force and $\omega_{d}$ is the angular frequency of the electrostatic driving force. The steady-state solution of driving mode vibration can be expressed as

(26) $$x\left(t\right)=A_{x}\cos\left(\omega_{d}t+\varphi_{x}\right),$$

where $A_{x}$ is determined by the classical AGC of the driving control loop; $\varphi_{x}$ is the phase lag between *x* displacement and driving force, and $\varphi_{x}=-\pi /2$ is usually obtained when the PLL is stable. Moreover, according to Equation (17), PLL also keeps the drive mode in a resonant state ($\omega_{d}={\tilde{\omega }}_{1}$, where $\gamma_{12}=0$).

Because $\sigma_{2}$ is infinitesimal, the displacement response on the *y*-axis caused by the excitation coupling acceleration $g_{xc}$ can be ignored. Moreover, the *Q* value of the DRG in this paper is relatively high, the damping coupling acceleration $g_{\tau_{yx}}$ is several orders of magnitude smaller than the quadrature coupling acceleration $g_{q}$, that is $g_{q}\gg g_{\tau_{yx}}$, so $g_{\tau_{yx}}$ can also be ignored [9]. Equation (26) is substituted into Equation (25), and the steady-state displacement of the sensing mode can be obtained as

(27) $$y\left(t\right)=\overset{\mathrm{In-phase}\;\mathrm{Signal}}{\overset{︷}{\underset{y_{\Omega }\left(t\right)}{\underset{︸}{A_{y\Omega }\cos\left(\omega_{d}t+\varphi_{y}\right)}}}}+\overset{\mathrm{Quadrature}\;\mathrm{Signal}}{\overset{︷}{\underset{y_{q}\left(t\right)}{\underset{︸}{A_{yq}\sin\left(\omega_{d}t+\varphi_{y}\right)}}}},$$

with

(28) $$\begin{array}{cc} A_{y\Omega } & =-\frac{2\nu \Omega A_{x}\omega_{d}}{\sqrt{{\left({\tilde{\omega }}_{yy}^{2}-\omega_{d}^{2}\right)}^{2}+{\tilde{\omega }}_{yy}^{2}\omega_{d}^{2}/Q_{yy}^{2}}}, \end{array}$$

(29) $$\begin{array}{cc} A_{yq} & =-\frac{\omega_{yx}^{2}A_{x}}{\sqrt{{\left({\tilde{\omega }}_{yy}^{2}-\omega_{d}^{2}\right)}^{2}+{\tilde{\omega }}_{yy}^{2}\omega_{d}^{2}/Q_{yy}^{2}}}, \end{array}$$

(30) $$\begin{array}{cc} Q_{yy} & =\tau_{yy}{\tilde{\omega }}_{yy}/2,\;\varphi_{y}=-\arctan\frac{\omega_{d}{\tilde{\omega }}_{yy}/Q_{yy}}{{\tilde{\omega }}_{yy}^{2}-\omega_{d}^{2}}, \end{array}$$

where $\varphi_{y}$ is the phase lag caused by the sensing mode; $y_{\Omega }\left(t\right)$ is the Coriolis response signal; $y_{q}\left(t\right)$ describes the response signal introduced by the stiffness coupling, which is quadrature to the phase of the Coriolis response signal, also known as the quadrature error.

From Equations (24), (28) and (30), when the mode-matching, that is, the resonance frequencies of the two modes are equal $\left(\omega_{d}={\tilde{\omega }}_{xx}={\tilde{\omega }}_{yy}={\tilde{\omega }}_{m1}={\tilde{\omega }}_{m2}={\tilde{\omega }}_{0}\right)$, then $\varphi_{y}=-90^{{}^{\circ}}$. This means that the phase lag information can be used to determine whether the two modes match, and at this time, the Coriolis displacement response of the DRG reaches the maximum value, so the mechanical sensitivity of the DRG can be expressed as

(31) $$S_{y\Omega m}=\left|\frac{A_{y\Omega }}{\Omega }\right|=\frac{2\nu A_{x}Q_{yy}}{{\tilde{\omega }}_{0}}.$$

#### 3.2.2. Control System Framework

**Figure 5:**
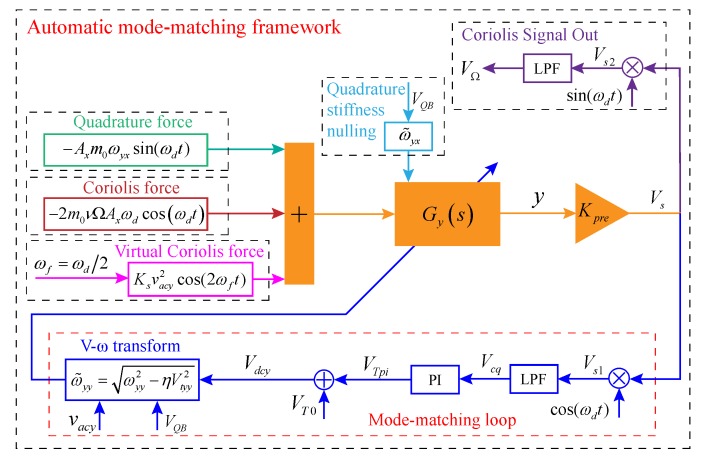
The automatic mode-matching control scheme framework.

The automatic mode-matching control scheme framework is shown in Figure 5. The excitation voltage $V_{y}=V_{dcy}+v_{acy}\cos\left(\omega_{f}t\right)$ or $V_{y}=V_{dcy}+v_{acy}\sin\left(\omega_{f}t\right)$ is applied to the excitation electrodes in the *y*-axis direction. $v_{acy}$ is the fixed amplitude value of the AC voltage. $V_{dcy}$ is the DC tuning voltage that is adjusted in real-time by a mode-matching loop. $\omega_{f}=\omega_{d}/2$ is the oscillation frequency of the AC voltage, which is half of the resonant frequency of the driving mode. Moreover, $\omega_{f}$ can be obtained by the Digital voltage controlled oscillator (DCO) module of the PLL in the driving mode. By substituting $V_{y}$ into Equation (10), ignoring the static force and the $\omega_{f}$ electrostatic force term, the virtual Coriolis force is obtained as

(32) $$f_{vc}=f_{y}=K_{s}v_{acy}^{2}\cos\left(2\omega_{f}t\right)=K_{s}v_{acy}^{2}\cos\left(\omega_{d}t\right),$$

where $\omega_{f}=\frac{1}{2}\omega_{d}$; $K_{s}$ is the conversion coefficients from voltage to force of the DRG. It acts on the sensing mode simultaneously with Coriolis and quadrature forces.

After that, the reference phase signals $\sin(\omega_{d}t)$ and $\cos(\omega_{d}t)$ are used for multiplication demodulation. Then various output signals of the sensing mode and phase measurement are obtained through Low Pass Filter (LPF). The phase metric reflecting the mode-matching state is taken as the control variable to control the tuning voltage in real-time. The frequency of the sensing mode is changed by the electrostatic negative stiffness effect to realize the automatic mode-matching.

#### 3.2.3. Analysis of Mode-Matching Loop

Due to $\sigma_{1}$ and $\sigma_{2}$ are infinitesimals, the response of the forces introduced by $2\sigma_{1}/m_{0}$ and $2\sigma_{2}/m_{0}$ to the *y*-axis displacement can be ignored. From the analysis in Section 3.2.1 above, $g_{\tau_{yx}}$ can also be ignored. According to the analysis of Equations (19) and (20), it is known that to achieve mode-matching, the stiffness coupling (quadrature error signal) must be eliminated first. Because the quadrature coupling stiffness between two modes is quasi-static, its change speed is extremely slow. Therefore, it is assumed that the quadrature error has been suppressed to a small degree or even to zero by the quadrature stiffness correction voltage $V_{QB}$ in advance. After quadrature stiffness nulling, the Coriolis response signal and the virtual Coriolis response signal can be obtained by multiplication demodulation. By substituting Equations (26) and (32) into Equation (25), combining the control block diagram shown in Figure 5, the vibration displacement response of the sensing mode is described as

(33) $$V_{s}=\overset{y_{\Omega }}{\overset{︷}{\underset{\mathrm{Coriolis}\;\mathrm{response}}{\underset{︸}{A_{y\Omega 1}\cos\left(\left(\omega_{d}+\omega_{\Omega }\right)t+\varphi_{y+\omega_{\Omega }}\right)+A_{y\Omega 2}\cos\left(\left(\omega_{d}-\omega_{\Omega }\right)t+\varphi_{y-\omega_{\Omega }}\right)}}}}+\overset{y_{vc}}{\overset{︷}{\underset{\mathrm{Virtual}\;\mathrm{Coriolis}\;\mathrm{response}}{\underset{︸}{A_{yvc}\cos\left(\omega_{d}t+\varphi_{y}\right)}}}},$$

where

(34) $$\begin{array}{cc} A_{yvc} & \approx -\frac{K_{s}v_{acy}^{2}K_{pre}/m_{0}}{\sqrt{{\left({\tilde{\omega }}_{yy}^{2}-\omega_{d}^{2}\right)}^{2}+{\tilde{\omega }}_{yy}^{2}\omega_{d}^{2}/Q_{yy}^{2}}}, \end{array}$$

(35) $$\begin{array}{cc} A_{y\Omega 1} & =-\frac{\nu \Omega_{c}A_{x}\omega_{d}K_{pre}}{\sqrt{{\left({\tilde{\omega }}_{yy}^{2}-{\left(\omega_{d}+\omega_{\Omega }\right)}^{2}\right)}^{2}+{\tilde{\omega }}_{yy}^{2}{\left(\omega_{d}+\omega_{\Omega }\right)}^{2}/Q_{yy}^{2}}}, \end{array}$$

(36) $$\begin{array}{cc} A_{y\Omega 2} & =-\frac{\nu \Omega_{c}A_{x}\omega_{d}K_{pre}}{\sqrt{{\left({\tilde{\omega }}_{yy}^{2}-{\left(\omega_{d}-\omega_{\Omega }\right)}^{2}\right)}^{2}+{\tilde{\omega }}_{yy}^{2}{\left(\omega_{d}-\omega_{\Omega }\right)}^{2}/Q_{yy}^{2}}}, \end{array}$$

(37) $$\begin{array}{cc} \varphi_{y} & =-\arctan\frac{\omega_{d}{\tilde{\omega }}_{yy}/Q_{yy}}{{\tilde{\omega }}_{yy}^{2}-\omega_{d}^{2}}=-\arctan\frac{2\omega_{d}/\tau_{yy}}{{\tilde{\omega }}_{yy}^{2}-\omega_{d}^{2}}, \end{array}$$

(38) $$\begin{array}{cc} \varphi_{y+\omega_{\Omega }} & =-\arctan\frac{2\left(\omega_{d}+\omega_{\Omega }\right)/\tau_{yy}}{{\tilde{\omega }}_{yy}^{2}-{\left(\omega_{d}+\omega_{\Omega }\right)}^{2}}, \end{array}$$

(39) $$\begin{array}{cc} \varphi_{y-\omega_{\Omega }} & =-\arctan\frac{2\left(\omega_{d}-\omega_{\Omega }\right)/\tau_{yy}}{{\tilde{\omega }}_{yy}^{2}-{\left(\omega_{d}-\omega_{\Omega }\right)}^{2}}, \end{array}$$

where $\varphi_{y+\omega_{\Omega }}$ and $\varphi_{y-\omega_{\Omega }}$ are the phase delay caused by the signal $\cos(\left(\omega_{d}+\omega_{\Omega }\right)t)$ and $\cos(\left(\omega_{d}-\omega_{\Omega }\right)t)$ through the sense mode. According to the phase-frequency characteristics at the resonant state, $\varphi_{y+\omega_{\Omega }}$ and $\varphi_{y-\omega_{\Omega }}$ are symmetric approximately $-90^{{}^{\circ}}$ in the mode-matching, that is, $\varphi_{y+\omega_{\Omega }}+\varphi_{y-\omega_{\Omega }}=-180^{{}^{\circ}}$. It can be noted that virtual Coriolis force for the sensing mode is in phase with Coriolis force, which will cause output offset $V_{\Omega =0}\approx y_{vc}$. It is easily subtracted from the sensing mode output.

To simplify the analysis, the input angular rate is set to a constant value, that is, $\omega_{\Omega }=0,\Omega =\Omega_{c}$. In Figure 5, then $V_{s}$ is demodulated by reference signals $\cos(\omega_{d}t)$ and $\sin(\omega_{d}t)$, and two signals $V_{s1}$ and $V_{s2}$ can be obtained as respectively

(40) $$\begin{array}{cc} V_{s1} & =\cos\left(\omega_{d}t\right)*\left\{A_{yvc}\cos\left(\omega_{d}t+\varphi_{y}\right)+A_{y\Omega }\cos\left(\omega_{d}t+\varphi_{y}\right)\right\},\\ V_{s2} & =\sin\left(\omega_{d}t\right)*\left\{A_{yvc}\cos\left(\omega_{d}t+\varphi_{y}\right)+A_{y\Omega }\cos\left(\omega_{d}t+\varphi_{y}\right)\right\}, \end{array}$$

where $A_{y\Omega }=A_{y\Omega 1}+A_{y\Omega 2}$. Moreover, in order to ensure the normal operation of the mode-matching loop, $|A_{yvc}\left|>\right|A_{y\Omega }|$ is generally required.

Then, the two demodulated signals are filtered by LPF to obtain as

(41) $$\begin{array}{cc} V_{cq} & =LPF\left\{V_{s1}\right\}=\frac{A_{yvc}}{2}\cos\left(\varphi_{y}\right)+\frac{A_{y\Omega }}{2}\cos\left(\varphi_{y}\right), \end{array}$$

(42) $$\begin{array}{cc} V_{\Omega } & =LPF\left\{V_{s2}\right\}=-\frac{A_{yvc}}{2}\sin\left(\varphi_{y}\right)-\frac{A_{y\Omega }}{2}\sin\left(\varphi_{y}\right). \end{array}$$

In addition, according to Equation (22), after quadrature stiffness nulling, the resonance frequency of the driving mode is converted from $\omega_{d}={\tilde{\omega }}_{1}$ to $\omega_{d}={\tilde{\omega }}_{xx}={\tilde{\omega }}_{m1}$, which can be controlled in real-time by the PLL. Then, Equation (41) can be simplified as

(43) $$V_{cq}=\frac{A_{yvc}}{2}\cos\left(\varphi_{y}\right)+\frac{A_{y\Omega }}{2}\cos\left(\varphi_{y}\right)=\left(\frac{A_{yvc}}{2}+\frac{A_{y\Omega }}{2}\right)\cos\left(\varphi_{y}\right).$$

From Equation (43), it can be seen that when the mode-matching ($\omega_{d}={\tilde{\omega }}_{xx}={\tilde{\omega }}_{yy}={\tilde{\omega }}_{m1}={\tilde{\omega }}_{m2}={\tilde{\omega }}_{0}$), then $\varphi_{y}=-\frac{\pi }{2}$ and $V_{cq}=0$. Therefore, $V_{cq}$ can be used as a basis for judging whether the mode-matching, which is also taken as the input of the proportional integral (PI) controller of the mode-matching loop. Moreover, the tuning voltage $V_{dcy}=V_{T0}+V_{Tpi}$ is controlled by setting the reference value as 0 to achieve the elimination of frequency split. Similarly, $V_{T0}$ is the preset voltage of the mode-matching real-time control loop, which can be obtained by manually adjusting the DC voltage applied to the *y*-axis excitation electrodes so that $V_{cq}=0$.

On the other hand, according to Equation (42), for the Coriolis output channel, when the mode-matching, then

(44) $$V_{\Omega }=-\frac{A_{yvc}}{2}\sin\left(\varphi_{y}\right)-\frac{A_{y\Omega }}{2}\sin\left(\varphi_{y}\right)=-\left(\frac{A_{yvc}}{2}+\frac{A_{y\Omega }}{2}\right)\sin\left(\varphi_{y}\right),$$

it can be seen that applying the virtual Coriolis force on the sensing mode will cause the output offset $V_{\Omega =0}=-\frac{A_{yxc}}{2}$, but it can be easily subtracted from the sensing output.

In addition, combining Equations (43) and (44), the actual phase delay of the sensing mode can be estimated by $\varphi_{y}=\arctan(V_{\Omega }/V_{cq}$). Then, according to Equation (37), the actual frequency split degree in the mode-matching state can be calculated.

## 4. Simulation Analysis for Automatic Mode Matching

In order to verify the feasibility of the closed-loop mode-matching control system and the effectiveness of the theoretical analysis, according to the principle shown in Figure 5, the system simulation is implemented by MATLAB SIMULINK. The main parameters of the DRG in the simulation system are shown in Table 2. The parameters in Table 2 are obtained by actual testing, which are used for system simulation. The other structural parameters are shown in Table 1.

Because quadrature stiffness correction is not the focus of this paper, it is only a brief introduction. Therefore, in this paper, the simulation analysis of the automatic mode-matching process is mainly carried out after the coupling stiffness (quadrature error) between two modes is suppressed to almost 0 by the quadrature stiffness correction voltage $V_{QB}$. In addition, the initial input angular rate Ω is zero.

**Figure 6:**
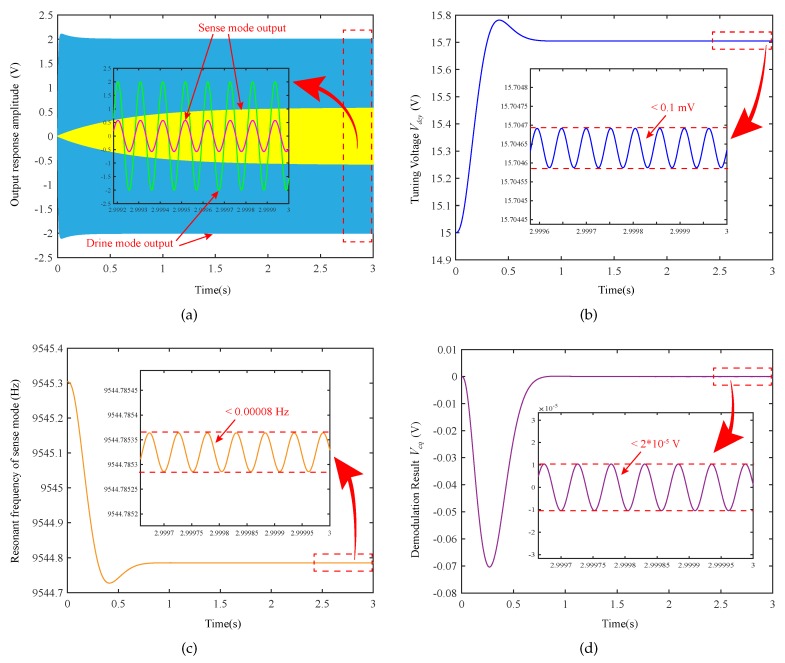
Automatic mode-matching process: (**a**) output response amplitudes ($V_{d}$ and $V_{s}$) of two modes; (**b**) tuning voltage $V_{dcy}$; (**c**) demodulation Result $V_{cq}$; (**d**) resonant frequency of sense mode $f_{yy}$ $\left(f_{yy}≜\omega_{yy}/\left(2\pi \right)\right)$.

The various waveforms of the automatic mode-matching process is shown in Figure 6. Figure 6a shows that the detection outputs of the driving mode and the sensing mode are $V_{d}$ and $V_{s}$, respectively. Moreover, the captured phase and amplitude information are displayed in Figure 6b by embedding the enlarged figure. It can be observed that when the designed mode-matching control works on the loop, the amplitude of the sensing mode increases sharply. When the phase between the two signals becomes matched, this means that it is now in a steady-state where the resonance frequency matching.

Figure 6b,c are the tuning voltage and resonance frequency of the sensing mode, respectively. As can be seen from Figure 6b, the preset voltage $V_{T0}=15.0$ V, the tuning voltage $V_{dcy}$ tends to be stable during mode-matching. The mode-matching is realized when the tuning voltage $V_{dcy}$ is 15.70 V, at this time, the two mode frequencies are 9544.79 Hz.

From Figure 6c, it can be seen that the electrostatic negative stiffness effect produced by the preset voltage $V_{T0}$ softens the resonant frequency of the sense mode in the initial stage. Moreover, since the resonant frequency of the driving mode is also affected by the electrostatic negative stiffness effect of the quadrature stiffness correction DC voltage and the driving excitation DC voltage, the resonant frequency of sensing mode after mode-matching is not equal to the frequency $f_{1}$ of the driving mode. The mode-matching frequency under electrostatic tuning will be smaller than its intrinsic resonance frequency, which is also in accordance with the analysis of Equation (24) above.

From Figure 6d, combined with the above analysis, $\varphi_{y}\approx -89.9995^{{}^{\circ}}$ can be calculated. Further, it can be calculated that the frequency split value during mode-matching is approximately $1.6\times 10^{-5}$ Hz.

**Figure 7:**
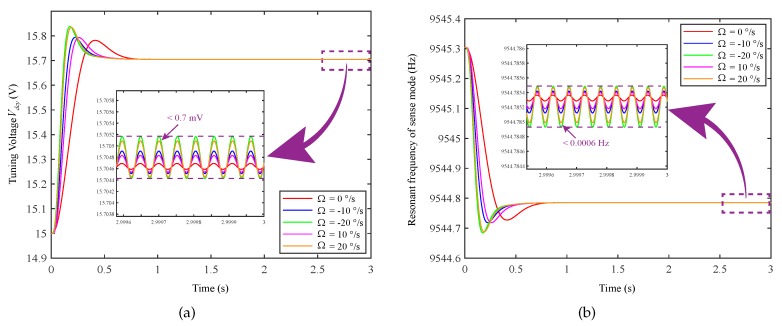
Influence of different input angular rates on tuning voltage and resonant frequency: (**a**) influence of different input angular rates on tuning voltage $V_{dcy}$; (**b**) influence of different input angular rates on resonant frequency $f_{yy}$ of sensing mode.

The effect of different input angular rates Ω on the tuning voltage and resonance frequency is shown in Figure 7. The interference fluctuation of the different Ω to the resonance frequency $f_{yy}$ is less than 0.0006 Hz. The interference fluctuation of the different Ω to the tuning voltage $V_{dcy}$ is less than 0.7 mV. This shows that when Ω exists, the frequency tuning system can still work normally and finally stabilize at the desired frequency.

**Figure 8:**
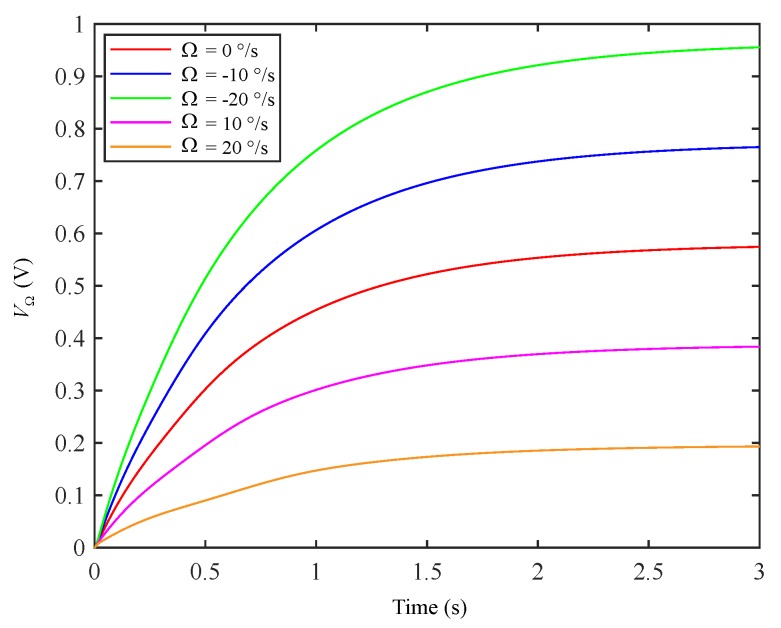
Coriolis signal output curves at different angular rates.

Considering that Ω is $0^{{}^{\circ}}$/s, $\pm 10^{{}^{\circ}}$/s, and $\pm 20^{{}^{\circ}}$/s, respectively, the corresponding Coriolis signal output curve is obtained, as shown in Figure 8. This indicates that the system can still detect the input angle rate when the mode-matching loop works normally. It can also be seen that applying a virtual Coriolis force on the sensing mode will cause the output offset ($V_{\Omega =0}\neq 0$), but it can be easily subtracted from the Coriolis channel of the sensing output.

## 5. Experimental Analysis Results

### 5.1. Experimental Equipment and Circuit

**Figure 9:**
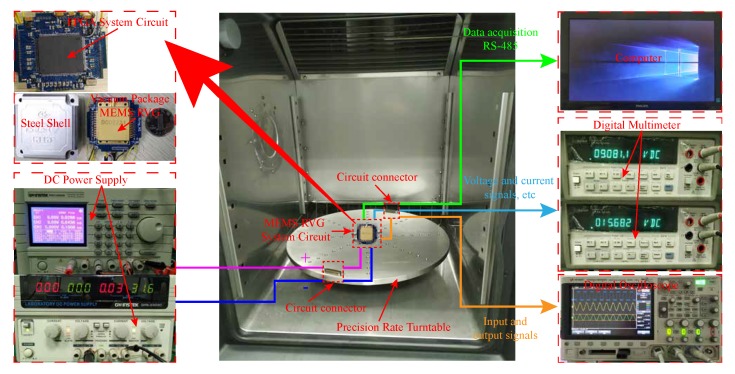
MEMS DRG circuit and experimental equipment.

In order to verify the effectiveness of the automatic mode-matching technology of the MEMS disk resonator gyroscope (DRG), the digital circuit of the gyroscope based on field-programmable gate array (FPGA) is designed and the relevant test experiments are carried out to verify the feasibility of the system. Taking DRG as the experimental object, the driving mode closed-loop, quadrature stiffness correction, and mode-matching control system of DRG are realized by using FPGA. Figure 9 shows the test equipment and the DRG system circuit. The test equipment mainly includes DC power supply, digital oscilloscope, digital multimeter, computer, and precision rate turntable. Two DC power supplies are used to provide DC voltage and ground to the gyroscope circuit system. The digital oscilloscope is used to observe the different input and output signals of the gyroscope. The digital multimeters are used to test the current and voltage signals of the circuit system. This computer is used to measure and process various data from the gyroscope. The scale factor of the gyroscope is measured by precision rate turntable.

**Figure 10:**
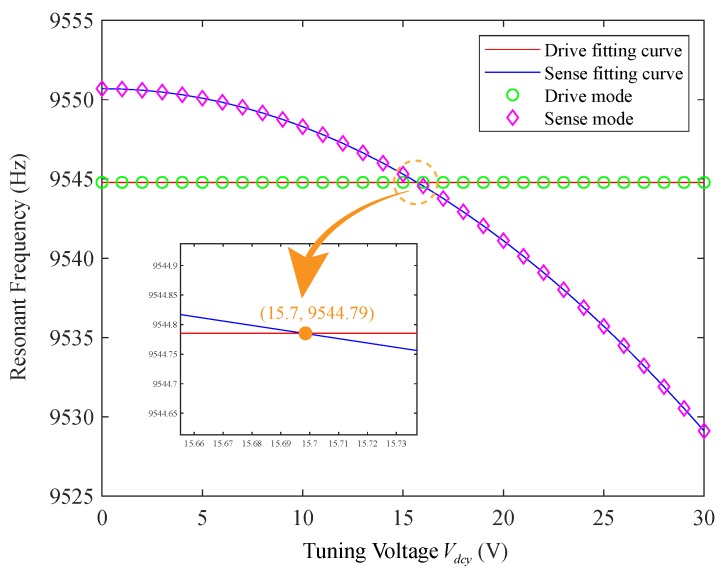
Relationship between two mode resonant frequencies and tuning voltage after quadrature stiffness nulling.

After the quadrature error nulling, the resonance frequencies of driving mode and sensing mode under different tuning voltages are measured by the sweep method on the studied gyroscope, as shown in Figure 10. Moreover, the relationship between resonance frequency and tuning voltage of the two modes is obtained and compared with the curve obtained by the least-square nonlinear fitting of the above theoretical formula by MATLAB. It can be seen from the figure that the actual data point trajectory is basically consistent with the theoretical fitting curve, which shows the correctness of the theoretical derivation.

### 5.2. Mode-Matching Implementation Process

**Figure 11:**
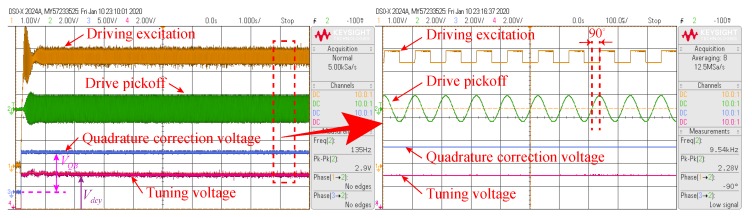
The waveforms of the mode-matching system startup process at room temperature.

The waveforms of the mode-matching system startup process at room temperature are shown in Figure 11. The quadrature error caused by structural asymmetry and process error has a significant influence on the bias performance of MEMS DRG. From the analysis of Equation (19) above, it can be seen that the suppression of quadrature error is the premise of mode-matching. Therefore, it is necessary to suppress the quadrature error by the quadrature stiffness correction voltage $V_{QB}$ ($V_{QB}=9.0811$ V) firstly.

**Figure 12:**
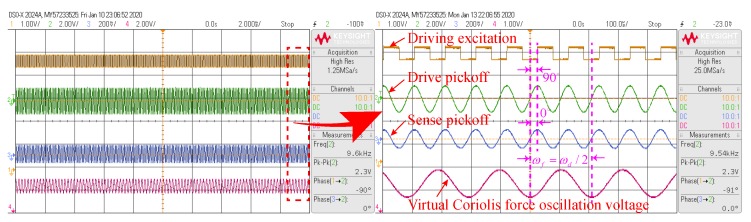
Phase relationship between signals under mode-matching steady state condition.

After the quadrature error signal is suppressed to approximately zero, an AC voltage with a fixed amplitude and a frequency that is half the driving mode resonance frequency is applied to the excitation electrode of the sensing mode to obtain a corresponding virtual Coriolis force. Then the mode-matching is judged by the phase difference between the virtual Coriolis force and the sensing mode detection output. The test results after mode-matching are shown in Figure 12.

It can be seen from Figure 12 that after the mode-matching, the phase difference between the drive excitation signal(In phase with the virtual Coriolis force signal) and the sense pickoff is about $-90^{{}^{\circ}}$. The phase difference between the driving pickoff and the sensing pickoff is about $0^{{}^{\circ}}$. That is to say, the drive pickoff signal and sensing pickoff signal are in the same phase, which is consistent with the previous theoretical and simulation analysis results. Moreover, it can also be seen that the voltage oscillation frequency for the virtual Coriolis force is half the resonant frequency of the driving mode. When the mode-matching is in steady-state, the tuning voltage ($V_{dcy}=V_{T0}+V_{Tpi}$) stabilizes at approximately 15.6820 V, which is basically consistent with the simulation result. Due to the influence of electrostatic negative stiffness effect, when the modes are matched, the resonance frequency of the driving mode is reduced to about 9544.79 HZ. The result is in accordance with the derivation of the Equation (24). Under the steady-state of mode-matching shown in Figure 12, the phase delay $\varphi_{y}\approx -88.95^{{}^{\circ}}$ can be calculated by $\arctan(V_{\Omega }$/$V_{cq})$. Then, according to the above analysis, it can be estimated that the frequency split under mode-matching is within about 0.1 Hz. When the modes are matched, there is a certain difference between the simulation and experimental results, which is mainly because the damping error is ignored in the simulation. In practical system, when the modes are matched, the damping error will become the main error factor of the DRG system. When the DRG works in the rate mode, the error introduced by the damping mismatch can be regarded as the in-phase error of the Coriolis force response of the sensing mode. When the Q value of the DRG is relatively high, this error can be approximately ignored here. The influence of the damping mismatch on the resonance frequency of the DRG can also be ignored.

Performance test results before and after mode-matching (room temperature, open-loop detection) are shown in Table 3. Open-loop detection of the DRG can be applied to low-rate input applications, such as gyrocompasses and inclinometers. In these applications, the effect of the rate on the mode frequency is small. In addition, at room temperature, the open-loop detection method is used to measure the scale factor of the DRG under the condition of mode mismatch and real-time mode-matching, and the performance of the DRG prototype is tested. The experimental results of the DRG show that the scale factor increases, the bias instability decreases, and the noise characteristics improve after the electrostatic tuning.

**Figure 13:**
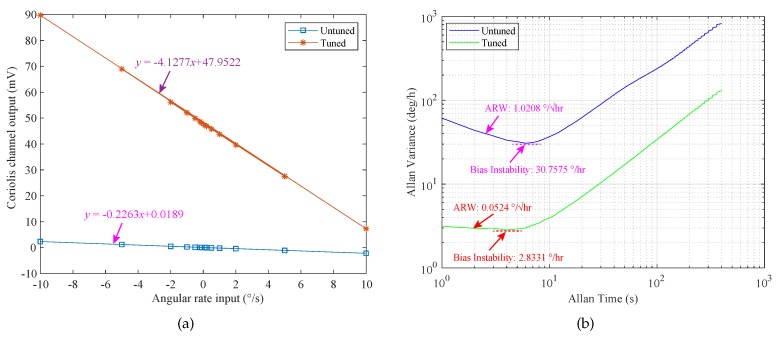
Scale factor and Allan variance curves under mode-matching and mode mismatch conditions: (**a**) the scale factor; (**b**) the Allan variance curves.

As shown in Figure 13a, in the case of mode mismatch and mode-matching, the scale factor of the DRG prototype is 0.2263 mV/${}^{{}^{\circ}}$/s and 4.1277 mV/${}^{{}^{\circ}}$/s, respectively. Then, the drift characteristics of the DRG prototype are evaluated at room temperature. The comparison of Allan variance curves under mode-matching and mode mismatch is shown in Figure 13b. Compared with the mode mismatch state, the bias instability of DRG under mode-matching is reduced from 30.7575 ${}^{{}^{\circ}}$/h to 2.8331 ${}^{{}^{\circ}}$/h, and the ARW is reduced from 1.0208 ${}^{{}^{\circ}}/\sqrt{h}$ to 0.0524 ${}^{{}^{\circ}}/\sqrt{h}$. Therefore, it can be seen that the ARW of the DRG is improved by 19.48 times by the automatic mode-matching control. The above experimental results show that reducing the frequency difference between the driving mode and the sensing mode through the mode-matching technology is beneficial to improving the mechanical sensitivity and bias stability or Allan deviation measurement of the MEMS DRG. However, the measurable range is $\pm 10^{{}^{\circ}}$/s, which is significantly reduced, because under the mode-matching condition, under the same angular rate input, the vibration displacement (Coriolis response) of the sensing mode is the largest. The measurable range is reduced due to the limitations of the DRG structure and detection circuit. However, this problem can be alleviated by the force feedback closed-loop detection method.

## 6. Conclusions

This paper proposes an automatic matching method for MEMS DRG based on virtual Coriolis force signal. This mode-matching method eliminates the need to design additional tuned electrodes separately, making structure design simple and reducing structural errors. By using the quadratic relationship between the driving voltage and the electrostatic force, a virtual Coriolis force is obtained by applying an AC voltage with a frequency that is half of the resonant frequency of the driving mode to the sensing electrode. The phase difference between the virtual Coriolis force and the sensitive output signal is used for modal matching. Firstly, the structural characteristics and electrode distribution of DRG are briefly introduced. In addition, the theories of DRG mode-matching and quadrature correction are studied in detail. Moreover, the design of the automatic mode-matching digital circuit control system is introduced. The system simulation is carried out by Simulink to verify the effectiveness of the mode-matching control system. The simulation results are basically consistent with the theoretical analysis. Then, the experimental results show that under the control of mode-matching at room temperature, the frequency split is controlled within 0.1 Hz. Compared with mode mismatch, the scale factor is increased, the bias instability is reduced, and the noise characteristics are improved. Due to the open-loop detection method was adopted in this study, the measurable range is small. Besides, for open-loop detection, during mode-matching, as the temperature increases, the *Q* value will decrease, which may lead to the reduction of mechanical sensitivity and the increase of noise, these problems can be solved by closed-loop detection. Nevertheless, the traditional force feedback closed-loop detection method cannot be used directly. Therefore, in future research, a feedback closed-loop detection method that matches the mode-matching method based on virtual Coriolis force will be constructed to improve the performance of the MEMS DRG further.

## Figures and Tables

**Table 1:** Structure parameter dimension of the DRG.

Parameter	Value	Unit
Radius of disk *r*	3000	μm
Thickness of nested ring *d*	120	μm
Width of nested ring *l*	80	μm
Single electrode width ϑ	20	${}^{{}^{\circ}}$
Initial electrode gap $h_{0}$	5.2	μm
Radius of anchor *R*	750	μm
Thickness of spoke st	8	μm

**Table 2:** The main parameters of the DRG in the simulation system.

Parameter	Value	Unit
Drive mode resonance frequency $f_{1}=\omega_{1}/\left(2\pi \right)$	9546.06	Hz
Driving mode quality factor $Q_{1}$	19,133	
Sense mode resonance frequency $f_{2}=\omega_{2}/\left(2\pi \right)$	9552.33	Hz
Sense mode quality factor $Q_{2}$	19,265	
Quadrature stiffness correction voltage $V_{QB}$	9.08	V
Mode matching loop preset voltage $V_{T0}$	15.0	V
Vacuum permittivity ϵ	$8.85\times 10^{-12}$	F/m
Mode effective mass $m_{0}$	$6.0\times 10^{-7}$	kg

**Table 3:** Performance test results (room temperature, open-loop detection).

Parameter	Mode Mismatch	Mode Matching
Frequency Split (Hz)	6.2700	<0.1000
Scale Factor (mV/${}^{{}^{\circ}}$/s)	0.2263	4.1277
Measurable Range (${}^{{}^{\circ}}$/s)	±10	±10
Bias Instability (${}^{{}^{\circ}}$/h)	30.7575	2.8331
ARW (${}^{{}^{\circ}}/\sqrt{h}$)	1.0208	0.0524
